# A Practical Treatise on Injuries of the Head

**Published:** 1831

**Authors:** 


A PRACTICAL TREATISE ON INJURIES OF THE HEAD. 12mo. pp. 121, Dublin, 1831.

THIS is really, of its kind, an useful volume, and is likely to be an acceptable one to young men. It is a compilation from all the authorities on the subject of injuries of the head, and the experience and opinions, the tenets and the modes of practice of Pott and Abernethy, Sir Astley Cooper and Mr. Brodie, Mr. Dease and Mr. Colles, will here be found concentrated and condensed. The Irish ought certainly to become acquainted with the nature and treatment of broken heads, and we are not a whit surprised that the present production should emanate from Dublin. The injuries of the scalp, skull, and brain are treated of in detail, and, after the consideration of each, we are presented with an epitome of the general remarks in the shape of a series of aphorisms. We think that our readers will not be displeased with a sight of a few of them.

### I. APHORISMS RESPECTING WOUNDS OF THE SCALP.

"Wounds of the scalp do not essentially differ from wounds of similar parts situated elsewhere, and are to be treated on precisely the same principles.

In the treatment of wounds of the scalp, you should have constantly in view its preservation.

Union by the first intention is always to be attempted in incised and lacerated flap wounds, not combined with fractures, &c.

If a scale of bone be cut off, and adhere to the flap, it makes no difference in the treatment. Proceed as if such complication did not exist.

In contused wounds, if small, approximate the parts, but by no means bring them into very close apposition.

Flap wounds which are much contused are to be treated by laying down the flap, after washing clean the surfaces. After the process of sloughing has taken place, bring the parts into the closest apposition.

Never interpose a dressing between the flap and skull.

Treat punctured wounds of the scalp as similar wounds in other parts, and like structures.

To remove the inflammatory tension of the aponeurosis produced by these wounds, and the consequent fever, dilate the puncture by incision.

Erysipelas, with fever, is not an unfrequent consequence of wounds of the scalp.

Always keep in mind the proximity of those wounds to the brain; the vascular connexion between the pericranium and dura mater; and the necessity, on this account, of a strict attention to the antiphlogistic regimen in the treatment."

We see nothing objectionable in the foregoing precepts; on the contrary they appear to be judicious and unexceptionable. We would observe, en passant, that in attempting to bring together the edges of a scalp-wound, in order to unite them by the first intention the surgeon should not apply strip over strip of sticking-plaister, as is usually done. The best plan is to use as few strips as possible, interlacing them in such a manner as to insure their firm adhesion, at the same time that intervals are left for the escape of matter, if it should be formed. The plaister should be changed, under ordinary circumstances, on the third day. Frequently the edges unite whilst some suppuration has taken place in the cellular membrane within. Break up the adhesions if the wound be small; a part of them in the most favourable situation if it be large, and allow the matter to escape. A poultice is to be applied to the wound, or to the part of it where the adhesions have been destroyed, and the remainder, in the latter case, is to be supported by a few adhesive straps. Such is the mode of treatment which we have found the best for such scalp-wounds.

### II. Aphorisms on Contusions of the Scalp.

"Contusions of the head are generally attended with detachment of the scalp from the pericranium, and a *bloody tumor* is the consequence.

The feel of this tumor so nearly resembles that of a depressed fracture, as often to be mistaken for it.

Never, on this account, make an incision in order to examine the state of the skull; but wait for the occurrence of those symptoms which render fracture more probable.

The application of cold lotions to the tumor, with the occasional use of laxative medicines, will, in general, be sufficient for the removal of the extravasated blood.

If these means should not be successful at the end of ten or twelve days, let out the blood by an incision."

We doubt the propriety of the latter piece of advice. We have seen more than one instance of the bloody tumour of the scalp remaining for upwards of a fortnight, and at length slowly yielding to lotions of muriate of ammonia, so that we should hesitate ere we plunged a lancet into the tumour. We have witnessed this practice, but suppuration occurred, and although so far as we remember, no bad consequences ultimately ensued, yet the general complexion of the case did not give us a favourable impression of its merits. The mass of practitioners, we fear, are not sufficiently acquainted with the characters of this bloody tumour of the scalp. We have seen two instances in which egregious blunders have been made.

### III. Aphorisms on Erysipelas of the Scalp.

"If, between the third and seventh day from the receipt of the wound, you perceive its lips to become puffy, and the surrounding integuments red and swollen, while at the same time symptoms of gastric disturbance make their appearance, you have reason to fear the occurrence of erysipelas.

The degree of danger of this disease is always proportioned to the violence of the fever, and recovery from the one is indicated by the cessation of the other.

In nine cases out of ten, the disease arises from disorder of the liver and other digestive organs, and to the removal of this disorder must your remedies be directed.

The constitutional treatment, in young and plethoric patients, consists in the use of blood-letting, emetics, purgatives, and other evacuants; followed, when the inflammation has subsided, and the tongue become clean, by tonics and nutritive diet.

In old, debilitated persons, the antiphlogistic treatment is contra-indicated, and recourse must be had to bark, &c. from the commencement.

The local treatment is always of less importance than the constitutional, and consists in the application of leeches, poultices, cold lotions, &c. to the wound and erysipelas.

On post-mortem examination of the brain of those who have died of erysipelas, no appearances indicative of disease of that organ can be detected."

Like all other accounts of erysipelas which we have read, this is abundantly leavened with sins both of omission and commission. Singularly enough the most characteristic symptom denoting the approach of erysipelas is not alluded to, we mean the rigor. How often have we seen this inspire a panic, and the secondary formations of matter have been apprehended when erysipelas only is to come. It is said that the degree of danger is commensurate with the violence of the fever. It is not so; the danger is dependent on the combination of many circumstances independent of violent fever. We are far from certain (and we have witnessed a great many cases of the disease) we are far from certain, we repeat, that in nine cases out of ten the disease arises from disorder of the liver and digestive organs. That the tongue is more or less foul, the intestinal secretions more or less depraved, we are willing to grant; but that these stand in the position of causes of the disease is more than we can venture to believe. We are certain also that the ordinary means of relieving disorder of the digestive organs will not cure a smart attack of erysipelas. The treatment in this town, particularly in our hospitals, though usually antiphlogistic in the first instance, need seldom be severe; bloodletting, for instance, not often being necessary. There are many, very many, considerations, into which we cannot enter at present, in the treatment of erysipelas. We repeat that we have never yet read a really good paper on the subject. Mr. Lawrence's is leather and prunella.

Talking of erysipelas of the scalp we cannot forbear noticing a much more severe affection; diffuse inflammation of the cellular membrane under the occipito-frontalis muscle and tendon. This is noticed, but clumsily, in the little work before us. Such as the description is we shall place it before our readers, not on account of its own merits, but of the paramount importance of a right understanding of the affection.

"These wounds are more likely than any we have as yet considered, to inflame and produce troublesome symptoms. This is generally true of all parts of the body, but in the scalp, particularly, they are sometimes attended with such a high degree of inflammation, and such dangerous symptoms, as to give cause for well-grounded alarm to both patient and surgeon. These symptoms, it is true, bear a close resemblance to those that occur from inflammation of any other part under fascia or aponeurotic structure; but, on account of the proximity of the inflammation to the brain and its membranes, they are peculiarly modified, and merit particular attention.

In this description of wounds, the inflammation which inevitably follows does not produce the same degree of tumefaction as we observe in erysipelas of the scalp; neither does it pit on pressure, which we shall presently see is another symptom of erysipelas. The swelling is generally of the natural colour of the skin, except in the immediate vicinity of the wound, where it is of a deep red colour, unmixed with the yellow hue which characterizes erysipelas. It is very tense, and extremely painful to the touch. In general, the ears and eye-lids are not comprehended in the tumour, although they may sometimes partake of the general inflammation of the skin, which occasionally attends those injuries.

The constitutional symptoms are usually extremely violent. Acute pain in the head, hot skin, parched tongue, excessive thirst, constipated bowels, high-coloured urine, restlessness, total want of sleep, very frequently delirium, with all the other symptoms of high inflammatory fever, are the almost constant attendants on punctured wounds penetrating the aponeurosis of the occipito-frontalis muscle. If the cause of those symptoms be not suspected, or the proper treatment not adopted, the patient's life is placed in the most imminent danger from the continuance of the fever; or, if he should fortunately escape a fatal termination of his sufferings, the injured aponeurosis and pericranium will become sloughy, abscesses will be produced, and the case rendered both tedious and troublesome.

How often do we see patients, under these formidable circumstances, ordered by their surgeons to have warm fomentations and emollient cataplasms applied to the wound, to the total neglect of the all-essential mode of practice! Do we not often see other surgeons, more considerate, prescribe the application of leeches to the tumor, or perhaps recommend a large quantity of blood to be taken from the arm, while the simplest and most efficient means of relieving the patient are entirely forgotten!

The mere enlargement of the wound, by a simple incision down to the bone, of an inch or less in length, will most commonly remove all the bad symptoms, and, if it be done in time, will render every thing else almost unnecessary. After having relieved the tension in this manner, if the inflammatory symptoms had previously run high, or if they should not be completely subdued by the operation, it will be proper to bleed from the arm, to an extent proportioned to the patient's age and strength, and to the violence of the symptoms; and afterwards make use of the other antiphlogistic remedies usually employed for the cure of inflammation, viz., purging, tartar-emetic in nauseating doses, strictly low diet, absolute rest, and the application of cold evaporating lotions to the head, or warm poultices, according to the sensations of the patient.

Desault is of opinion that all those symptoms may generally be removed by the exhibition of tartar-emetic. This is a dangerous error, and may lead to a fatal neglect of the practice here inculcated, which, by the greater number of intelligent surgeons, is considered indispensable in inflammation under the aponeurosis.

In punctured wounds of the scalp, perhaps the best practice would be, in all cases, even before the occurrence of inflammation, to enlarge the wound by a simple incision, and thus leave a free space for the tumefaction of the integuments and aponeurosis which almost always succeeds."

In the preceding observations there is something of good, and much of bad. In the first place, it is a mistake to imagine that diffuse inflammation of the cellular membrane of the scalp is exclusively produced by punctured wounds. We have seen it follow wounds of all descriptions, though it seems to be most common after those of a lacerated kind. The obvious deduction from this fact is, that enlarging a punctured wound, previous to the supervention of inflammation, is not likely to ward off the consequences which are dreaded. We have done this and we have seen it done, but we are far from satisfied of the utility of the practice. The description of wound to which it appears to be most applicable, is that in which there is a great contusion of the cellular membrane beneath the skin and aponeurosis, with a small punctured opening in the latter. When inflammation has occurred there can be no question of the propriety of incisions down to the pericranium. But here we dissent in toto from the position assumed by the author of this little work, that "the mere enlargement of the wound by a simple incision down to the bone, of an inch or less in length, will, most commonly, remove all the bad symptoms." We know, from experience, that in a London hospital it will not. Free incisions, we repeat, are required, and short of those, there is usually no safety for the patient. From the quotation which we have made, our readers would be led to imagine, that when diffuse inflammation has attacked the cellular membrane of the scalp, active blood-letting is necessary. Again we affirm, that in the London hospitals such treatment would be destruction, for the cellular membrane readily sloughs, and active depletion renders it still more disposed to do so. In some cases we have seen bark and incisions go hand in hand. In the great majority, antimonials and neutral salts, with occasional calomel purges are all that are required, independent of incisions. It is stated that in this affection there is little or no discoloration of the skin. This is a mistake. There is always, or has been so in the cases which we have witnessed, a blush of greater or less distinctness on the cutis of the scalp, and, more than that, this blush very frequently runs into common erysipelas. On this point, as on many others, we fear that the author of the little volume has drawn from books, rather than from life. We might make some further observations had we space and inclination. We are convinced that the common descriptions of erysipelas and diffuse inflammation of the scalp are vague and erroneous. With the following passage we must conclude.

### IV. Aphorisms on Suppuration within the Cranium.

"Acute inflammation of the brain, as a consequence of wounds or other injuries, resembles in its symptoms idiopathic phrenitis, and requires precisely the same treatment.

If, between the seventh and twenty-first day, irregular rigors, frequently recurring, and attended with fever, make their appearance, we have reason to dread the formation of matter within the cranium.

Suppuration within the cranium is sometimes caused by apparently trivial injuries of the head. The probability of its occurrence cannot be estimated by the extent of the injury.

The matter is much oftener situated within the dura mater, than between this membrane and the bone. It is generally smeared over the whole surface of the pia mater of one hemisphere.

If the matter be situated between the dura mater and skull, the patient will often recover by the timely application of the trephine; but, if the brain or pia mater be affected with suppuration, he will most probably die.

It will be time enough to use the trephine, when by irregular shiverings, &c. we have every reason to apprehend matter to be formed under the cranium.

Never apply the trephine as a means of *preventing* inflammation and suppuration of the brain or its membranes.

After every wound of the head, vigorous antiphlogistic treatment should at once be had recourse to, with a view to prevent suppuration within the cranium.

Abscesses in the liver occasionally follow wounds of the head. No satisfactory explanation of this fact has been given."

We have one observation to make. If the trephine is not applied till we are satisfied by "irregular shiverings," that is, by *many* shiverings that matter has been formed within the cranium, the probability is, that the operation will do no good. The trephine should be applied on the occurrence of the very first rigor, if local circumstances give any clue to the situation of the matter within. We cannot dismiss the small volume before us, without expressing our approbation of it as a whole, and our opinion that it is likely to be useful to the student and the practitioner. At the same time we cannot blind our eyes to its defects, nor refrain from cautioning the inexperienced portion of our readers against placing implicit confidence in all that is asserted in it.

